# Surgical Management of Falcotentorial Junction Tumors: A Case Series Report

**DOI:** 10.3389/fonc.2022.866225

**Published:** 2022-05-10

**Authors:** Peixi Liu, Xiaowen Wang, Yingjun Liu, Jiajun Cai, Zixiao Yang, Kai Quan, Wei Zhu, Jianping Song

**Affiliations:** ^1^Department of Neurosurgery, Huashan Hospital, Shanghai Medical College, Fudan University, Shanghai, China; ^2^National Center for Neurological Disorders, Fudan University, Shanghai, China; ^3^Shanghai Key Laboratory of Brain Function and Restoration and Neural Regeneration, Fudan University, Shanghai, China; ^4^Neurosurgical Institute, Fudan University, Shanghai, China; ^5^Shanghai Clinical Medical Center of Neurosurgery, Fudan University, Shanghai, China

**Keywords:** falcotentorial, hemangiopericytoma, meningioma, surgery, hybrid operation

## Abstract

**Objective:**

The surgical strategy for falcotentorial junction tumors remains complex. Different approaches are selected according to the location and growth pattern of the tumor and the operator’s experience. This report reviews our single-institution experience in the surgical management of falcotentorial junction tumors.

**Methods:**

We retrospectively reviewed the clinical and imaging data, surgical strategy, and follow-up outcomes of 49 patients treated from 2007 to 2020.

**Result:**

All 49 patients (12 male, 37 female, mean age: 56.3 ± 11.3 years) underwent safe tumor resection. The most common complaints were headache (43%), dizziness (39%), and unstable gait (16%). Thirty percent of the tumors showed calcification, and the computed tomography scans revealed hydrocephalus in 36% of the patients. On magnetic resonance imaging, 43% of the tumors were unilateral. According to the Asari classification, the tumors were divided into inferior (16%), superior (29%), anterior (22%), and posterior (33%) types. The occipital interhemispheric approach (88%) and supracerebellar–infratentorial approach (10%) were primarily used to reach the tumors. The pathology examination results revealed that 85.7% of the tumors were meningioma and 14.3% were hemangiopericytoma. Of the 49 patients, 15 achieved a Simpson grade I resection, and 29 achieved a Simpson grade II resection. The follow-up rate was 77.6% (38/45); 94.7% of patients (36/38) achieved a favorable outcome, and 9 experienced tumor recurrences.

**Conclusion:**

Surgical approach selection depends on the growth characteristics of the tumor and the degree of venous or sinus involvement. The occipital interhemispheric approach is the most commonly used and safest approach for falcotentorial junction tumors with multiple brain pressure control assistance techniques.

## Introduction

Falcotentorial junction tumors are located at the anterior portion of the junction between the falx and the tentorium (1–3). Rare falcotentorial junction tumors, such as meningiomas or hemangiopericytoma, are substantial surgical challenges due to their deep location and the presence of adjacent critical neurovascular structures. Different approaches can be selected according to the location and growth pattern of the tumor and operator preference (3–10). This study reviews our center’s experience in the surgical management of falcotentorial junction tumors.

## Methods

### Patient Cohort

The authors retrospectively reviewed the medical charts from our hospital’s main campus from June 2007 to April 2020 and roughly identified a total of 131 clinical cases of potential falcotentorial junction tumors. By carefully reviewing the clinical data and radiological images and excluding “peritorcular” tumors with torcular involvement or velum interpositum meningiomas (1–3, 11, 12), 49 patients with comprehensive medical data were enrolled in this study. The institutional review board of the authors’ hospital approved this study. The STROBE guidelines for observational cohort studies were followed. Due to the retrospective nature of the study, informed consent was waived.

The age, sex, clinical presentation, radiological findings, tumor location, surgical details, pathology, postoperative complications, surgical outcome, and follow-up data of the patients were collected and analyzed. The classic Asari classification, including inferior, superior, anterior, and posterior typing, was used to define the tumor location (13). There were four types. The anterior type means the tumor extension between the inferior sagittal sinus and the great vein of Galen). The inferior type means the tumor extension between the great vein of Galen and the straight sinus. The posterior type means the tumor extension along the straight sinus, and the superior type means the tumor extension above the cerebellar tentorium.

The Simpson grade was used to assess the extent of the resection. CT scanning was completed at 1 month after surgery. The MRI contrast was completed at 6 months after surgery. After that, if there was nothing special, a follow-up MRI contrast was required annually. Patient follow-up was conducted at the outpatient clinic or by telephone interview, and the recurrence rate and outcome were assessed. The modified Rankin Scale (mRS) score was used to quantify the outcome, in which a favorable outcome was defined as mRS ≤2.

### Literature Review

The authors performed a literature search on PubMed for articles on falcotentorial junction tumors published in English in the last 10 years. The search strategy was as follows: “(falcotentorial meningioma) OR (pineal region meningioma) [Allfield]”. A total of 68 results were retrieved; after reading their full texts, seven articles describing case series reports (more than 5 cases) were included for the literature review.

### Statistical Analysis

Statistical analysis was performed using SPSS 22.0 (IBM, USA). Numerical variables are presented as the mean ± standard deviation (range). A *p*-value <0.05 was set as the threshold of significance.

## Results

### Clinical Characteristics

The clinical characteristics of all 49 patients with falcotentorial junction tumors are summarized in **Table 1** (more detailed information can be found in the **Supplementary Table**). There were 12 males (24.5%) and 37 females (75.5%) in our series, aged 33 to 81 years (mean, 56.3 ± 11.3 years). The chief complaints were nonspecific symptoms such as headache (*n* = 21, 42.9%) and dizziness (*n* = 19, 38.8%), followed by unstable gait (*n* = 8, 16.3%), blurred vision (*n* = 5, 10.2%), limb weakness (*n* = 4, 8.2%), facial numbness (*n* = 2, 4.1%), and seizures (*n* = 1, 2.0%). Some lesions were found accidentally (*n* = 6, 12.2%).

**Table 1:** Clinical characteristics of patients with falcotentorial junction tumors.

Clinical characteristics	Value
Age (mean)	56.3 ± 11.3 years
Sex	
Male	12 (24.5%)
Female	37 (75.5%)
Clinical presentation	
Headache	21 (42.9%)
Dizziness	19 (38.8%)
Unstable gait	8 (16.3%)
Accidentally found	6 (12.2%)
Blurred vision	5 (10.2%)
Limb weakness	4 (8.2%)
Others	3 (6.1%)
Laterality	
Unilateral	21 (42.9%)
Bilateral	28 (57.1%)
Asari types	
Anterior	11 (22.5%)
Inferior	8 (16.3%)
Posterior	16 (32.7%)
Superior	14 (28.6%)
Straight sinus occlusion	
Yes	39 (79.6%)
No	10 (20.4%)
Preoperative GOS ≥4	49 (100%)
Surgical approach	
Occipital interhemispheric approach	43 (87.8%)
Supracerebellar–infratentorial approach	5 (10.2%)
Subtemporal approach	1 (2.0%)
Surgical results (Simpson grade)	
Simpson I	15 (30.6%)
Simpson II	29 (59.2%)
Simpson III	1 (2.0%)
Simpson IV	4 (8.2%)
Major postoperative complications	
Hydrocephalus	2 (4.1%)
Brain contusion	2 (4.1%)
Pathology	
Meningioma	42 (85.7%)
Fibrous type, WHO grade I	28 (57.1%)
Epithelial type, WHO grade I	12 (24.5%)
Angiomatous type, WHO grade I	1 (2.0%)
Transitional type, WHO grade I	1 (2.0%)
Hemangiopericytoma	7 (14.3%)
WHO grade I	1 (2.0%)
WHO grade II	3 (6.1%)
WHO grade III	3 (6.1%)
Follow-up	38 (77.6%)
Recurrence	9 (23.7%)
mRS ≤2	36 (94.7%)
mRS >2	2 (5.3%)^a^

mRS, modified Rankin Scale; WHO, World Health Organization.

a Including case 18 who passed away naturally in year 2015.

All patients were preliminarily diagnosed with meningioma preoperatively. The computerized tomography (CT) results showed 15 patients (30.6%) with calcification inside the tumor and 18 (36.7%) with hydrocephalus. On magnetic resonance imaging (MRI), the tumor was unilateral in 21 patients (42.9%) and bilateral in 28 (57.1%). According to the Asari classification, 8 tumors (16.3%) were of the inferior type, 14 (28.6%) were of the superior type, 11 (22.5%) were of the anterior type, and 16 (32.7%) were of the posterior type. The magnetic resonance venography (MRV) revealed a straight sinus occlusion rate of 79.6%.

### Surgical Strategy

**Figure 1:**
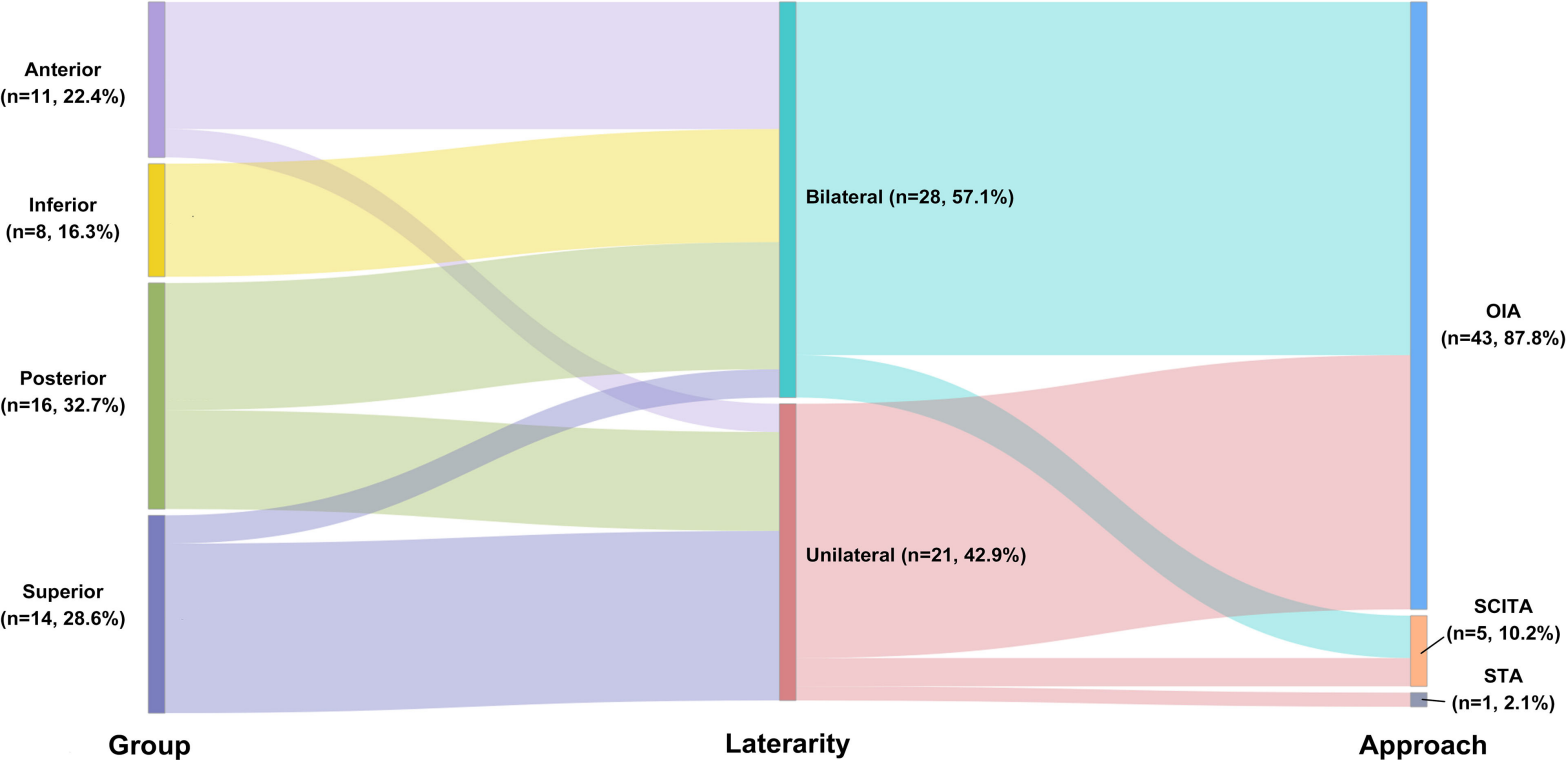
Sankey diagram showing the relationship among location, Asari type, and surgical approach selection.

In terms of surgical approach selection, the occipital interhemispheric approach (OIA, *n* = 43 87.8%) and supracerebellar–infratentorial approach (SCITA, *n* = 5, 10.2%) were mainly used. Only 1 tumor was resected by the subtemporal approach (2.0%, **Figure 1**). Three patients (12.2%; cases 6, 46, and 47) underwent preoperative external ventricular drainage, and 1 patient (2.0%, case 48; **Figure 3**) underwent preoperative tumor embolization.

### Surgical Outcome and Follow-Up

Gross total resection (Simpson grades I and II resection) was achieved in 44 (89.8%) surgeries; Simpson grades III and IV resections were achieved in 1 (2.0%) and 4 (8.2%) surgeries, respectively. For those patients with a diagnosis of hemangiopericyma, preoperative embolization may be helpful to reduce the blood supply in these cases. We have adopted such a strategy in recent clinical practice. All the patients with WHO II or beyond accepted radiotherapy after operation. The major postoperative complications were hydrocephalus treated by a V–P shunt (*n* = 2, 4.1%) and visual field impairment caused by contusion (*n* = 2, 4.1%). The pathology examination results confirmed a diagnosis of meningioma for 42 patients (85.7%) and hemangiopericytoma for 7 patients (14.3%). The mean follow-up time was 82.1 ± 39.9 months (15 to 169 months). The follow-up rate was 77.6% (38/49). Nine patients (23.7%) were found to have tumor recurrence in these followed-up patients. The pathology for 2 patients was hemangiopericytoma (WHO II), and the other 7 patients had meningioma (WHO I). No case reached Simpson I resection during the operation. Five cases reached Simpson II resection, and 1 case reached Simpson III resection. The other 3 cases were Simpson IV during operation. In total, 94.7% cases (36/38) achieved a favorable outcome.

### Literature Review

In the retrieved literature, the OIA or its modifications (occipital transtentorial approach, parieto-occipital, occipito-suboccipital) were the preferred approach for falcotentorial junction tumors (85.7%). Visual field impairment and hydrocephalus requiring shunting were the major surgical complications. The pathology examination results typically led to a diagnosis of meningioma, and most of the patients achieved a favorable outcome.

### Case Illustration

#### Case 1

**Figure 2:**
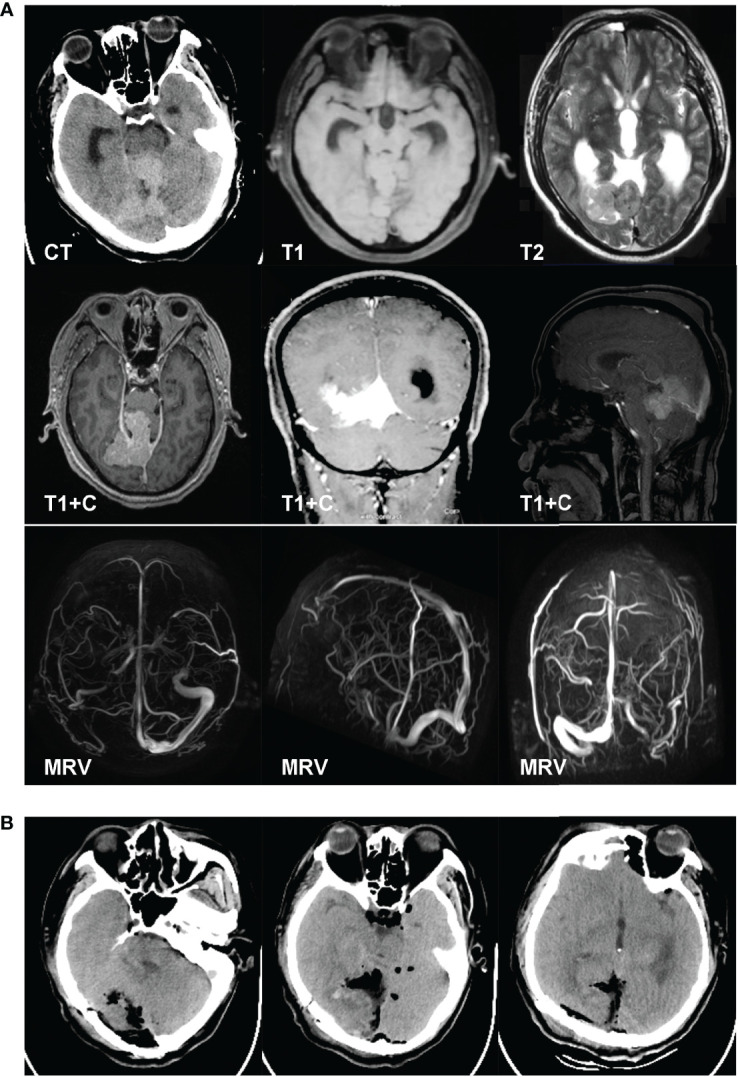
A 52-year-old man complained of blurred vision and unsteady gait for 2 months. The physical examination results showed left hemianopsia, and both VOS and VOD were 0.1. The MRI results revealed a falcotentorial meningioma, which had an isointense signal on T1-/T2-weighted imaging and homogeneous enhancement on contrast-enhanced MRI. The magnetic resonance venography results showed that the straight sinus was occluded **(A)**. We implanted an Ommaya reservoir at 1 week before tumor resection and maintained the drainage at 200 ml per day. The patient then underwent tumor resection using the occipital interhemispheric approach. During the operation, the majority of the tumor base was located at the falx end and the tentorium. The tumor was removed in a piecemeal fashion. Finally, the tumor and the base were totally removed (Simpson grade I resection). The postoperative CT showed no hemorrhage or infarction **(B)**. The pathology examination results revealed that the tumor was a hemangiopericytoma, WHO grade I.

A 52-year-old man complained of blurred vision and unsteady gait for 2 months. The physical examination showed left hemianopsia, and both left and right vision were 0.1. The MRI results revealed a falcotentorial meningioma, which had an isointense signal on T1-/T2-weighted imaging and homogeneous enhancement on contrast-enhanced MRI. The MRV showed that the straight sinus was occluded (**Figure 2A**). We implanted an Ommaya reservoir at 1 week before tumor resection and maintained drainage at 200 ml per day. The patient then underwent tumor resection using the OIA. During the operation, the majority of the tumor base was located at the falx end and the tentorium. The tumor was removed in a piecemeal fashion. Finally, the tumor and the base were totally removed (Simpson grade I resection). The postoperative CT showed no hemorrhage or infarction (**Figure 2B**). The pathology examination result revealed that the tumor was a hemangiopericytoma, WHO grade I. The 26-month follow-up showed no tumor recurrence.

#### Case 2

**Figure 3:**
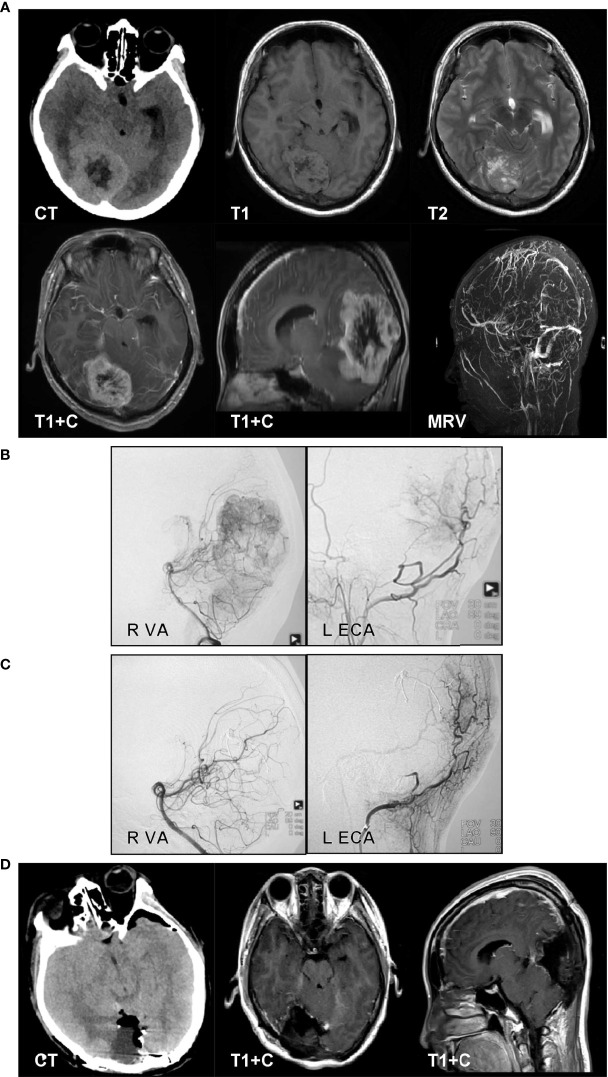
A 33-year-old man complained of headache for 2 months. The CT results revealed a mixed-density, right occipital lesion. The MRI results revealed a right occipital, mixed-signal lesion and flow voids on T1- and T2-weighted imaging. The lesion showed obvious enhancement on contrast-enhanced MRI. The magnetic resonance venography results showed an obstructed sagittal sinus **(A)**. We performed the operation in a hybrid operating room. The cerebral digital subtraction angiography (DSA) results revealed abundant tumor blood. The feeding arteries were the right posterior cerebral artery (PCA), right external carotid artery (ECA), right meningiohypophyseal trunk, and left ECA **(B)**. Onyx-18 was used for feeding artery embolization for the right PCA, right occipital artery, and left occipital artery. The DSA reexamination results showed that 90% of the blood supply was embolized **(C)**. Craniotomy was then performed. The occipital interhemispheric transtentorial approach was used to reach the tumor under navigation. The tumor was tenacious, with partial calcification. Piecemeal removal was performed, and we achieved a Simpson grade II resection. The postoperative CT results showed no hemorrhage or infarction **(D)**. The pathology examination results revealed that the tumor was a hemangiopericytoma.

A 33-year-old man complained of headache for 2 months. The CT results revealed a mixed-density, right occipital lesion. The MRI revealed a right occipital, mixed-signal lesion and flow voids on T1- and T2-weighted imaging. The lesion showed obvious enhancement on contrast-enhanced MRI. The MRV showed an obstructed sagittal sinus (**Figure 3A**). We performed the operation in a hybrid operating room. The cerebral digital subtraction angiography (DSA) results revealed abundant tumor blood supply. The feeding arteries were the right posterior cerebral artery (PCA), right external carotid artery (ECA), right meningiohypophyseal trunk, and left ECA (**Figure 3B**). Onyx-18 was used for feeding artery embolization for the right PCA, right occipital artery, and left occipital artery. The DSA reexamination showed that 90% of the blood supply was embolized (**Figure 3C**). Craniotomy was then performed. The occipital interhemispheric transtentorial approach was used to reach the tumor under navigation. The tumor was tenacious, with partial calcification. Piecemeal removal was performed, and we achieved a Simpson grade II resection. The postoperative CT results showed no hemorrhage or infarction (**Figure 3D**). The pathology examination results revealed that the tumor was a hemangiopericytoma, and the 10-month follow-up showed no tumor recurrence.

## Discussion

### Relationship Between the Tumor Growth Pattern and Approach Selection

MRI and angiography can provide abundant information on the location of the lesion and adjacent vascular structures, and the Asari classification can be used to define the tumor extension into four tiers according to the MRI findings (13). The choice of surgical approach depends on the relationship between the tumor, the deep venous system, and the tentorium (1, 13). The Asari classification gives us an imaging classification according to tumor extension. In most studies, OIA or one of its modifications was selected as the primary approach for falcotentorial junction tumors (**Figure 1** and **Tables 1**, **2**) (6, 12, 14, 15). Routine OIA was quite suitable for tumors that originated from the falx (similar to the superior Asari type) immediately above the junction of the vein of Galen with a straight sinus, especially if the tumor displaced the galenic venous system inferiorly. Parietal extension of the OIA (parietooccipital approach) can be performed for the posterior group, and transtentorial maneuver can be added for the anterior, inferior, and posterior groups.

**Table 2:** Literature review and summary of previously published studies on pineal meningioma.

Authors and year	Number of operations	Age (years)	Sex (M/F)	Tumor type (number)	Surgical approach (number)	Extent of resection	Pathology	Surgical-related complication	Hydrocephalus	Follow-up (month)	Recurrence	Outcome
Li, et al., 2011 (14)	10	53.3	4/6	Infratentorial > supratentorial (3)	Poppen (10)	GTR (6)	Meningioma	Intracranial infection (1) Intraventricular hemorrhage and pneumocephalus (1)	9/10 Relieved 1/10 VP shunt	14 (6–24)	No	KPS >70 (10)
				Supratentorial > infratentorial (2)			WHO I, (8); atypical WHO II(2)					
				Supratentorial = infratentorial (3)								
				Infratentorial (2)								
Nowak et al., 2014 (12)	6	52	1/5	Superior (5) Inferior (1)	Poppen (6)	GTR (2) (Simpson I: 1, II: 1) NTR (4) (Simpson III: 4)	Meningiomas	Temporary homonymous hemianopsia (5)	Pre-op: 1/3VP shunt, 1/3 ETV Post-op: 1/3 VP shunt	102 (24–160)	No	KPS >70 (6)
								Postoperative intraventricular hemorrhage (1)				
								Transient hemiparesis (1) Upward-gaze palsy (1)				
Qiu et al., 2014 (15)	15	51.3	5/10	Bassiouni’s classification Type I (7) Type II (4) Type III (4)	Poppen (15)	GTR (ll) NTR (3) STR (l)	Meningiomas Endotheliomatous (4) Fibrous (3) Transitional (3) Angioblastic (2) Psammomatous (1)	Homonymous visual deficit (2)	6/7 Relieved 1/7 VP shunt	28.3 (12–50)	No	NR
Chang et al., 2016 (6)	11	52.9	5/6	Asari classification Anterior (4), superior (2) Inferior (4), posterior (1) Bassiouni’s classification Type I (4), type II (4) Type III (2), type IV (1)	Poppen (8) Parieto-occipital (2) Occipital (1)	GTR (10) (Simpson I: 10) STR (1) (Simpson IV: 1)	Meningiomas Meningothelial (4) Fibrous (1) Transitional (4) Atypical (2)	Homonymous hemianopsia (2) Diplopia (1) Occipito-cerebellar hemorrhage (1) PCA infarction (1)	2/11 Relieved 1/11 VP shunt	35 (3–150)	No	Last follow-up KPS >70 (10) KPS <70 (1)
Tal acc hi et al., 2018 (5)	28	59.8	10/18	Supertentorial (12) Infratentorial (11) Supra-infratentorial (5)	Median suboccipital (11) Parieto-occipital and occipital (12) Suboccipital (5)	GTR (13) (Simpson I: 5, II: 8) STR (15) (Simpson III: 7, IV: 8)	Meningiomas (28)	Died of intractable cerebral edema (1) Tetraparesis (1), IV Cranial nerve deficit (1) Tumor bed hematoma requiring evacuation (2) Cerebrospinal fluid fistulae (2), Surgical wound infection (1)	Pre-op 9/28 Relieved Post-op 1/28 VP shunt	57.6	2/28 atypical	12 months follow-up KPS >70 (18) KPS<70 (4) Lost (6)
Joham Choque-Velasquez et al., 2019 (4)	10 (10/76)	NR	NR	NR	SCIT (N.A.) Occipital interhemispheric (N.A.)	GTR (9), PR (1)	Meningiomas (9) Anaplastic meningioma (1)	Thalamic infarction and died 1 month after surgery (1)	NR	94.5 (1–205)	No	Last follow-up 50% overall survival rate
	17	59.8	1/13	Asari classification								mRS	
	(14 patients)			Anterior (2), superior (4)	Poppen (4) SCIT (4)	Simpson I (4)		Visual field defects (2 permanent, 4 transient)	Pre-op:		3/17	Improved (4)	
Zhao et al., 2019 (3)				Inferior (9), posterior (1)	AIH (3) Torcular (2)	Simpson II (1)	Meningioma	Hemiparesis (2)	3/17 EVD, 1/17 ETV	29 (2–72)	(same patient)	Unchanged (9)	
				Bassiouni’s classification type I (1), type II (9), type III (4), type IV (2)	Transventricular (1) Staged Poppen + SCIT (3)	Simpson III (3) Simpson IV (9)	WHO I (9), WHO II (5)	Hemidysesthesia (1) Cerebellar hematoma (1) Hydrocephalus (1)	Post-op: 4/17 Resolved 1/17 VP shunt			Worsened (4)	

NR, not reported; SCIT, supracerebellar infratentorial approach; AIH, anterior interhemispheric transsplenial approach; GTR, gross total resection; NTR, near-total resection; STR, subtotal resection; PR, partial resection., N.A., not applicable.

We believe that the key point regarding the selection of surgical approach is whether the straight sinus is occluded. Theoretically, if an occluded straight sinus is observed on MRV, the OIA can be applied for all four Asari groups, supplemented by transtentorial maneuver (8, 16). Ergonomics may be the reason that neurosurgeons preferred the OIA. However, an unobstructed straight sinus should not be occluded under any circumstances. Although the SCITA was also suggested and might lower the chance of severe deep neurovascular compromise, the need for a semi-seated patient position might increase the risk of air embolism and surgeon fatigue. The author preferred to apply the SCITA only if the tumor was classified into the anterior or the inferior group, without a straight sinus occlusion, and/or the Galenic venous system was elevated. Bleeding from an injured straight sinus might be an issue for hemostasis with SCITA. Currently, the authors use a “head-up” park bench position for the SCITA to lower the risk of air embolism and improve the surgeons’ ergonomics and bleeding control (17). Regardless of the choice of surgical approach, the galenic venous system and collateral circulation should be preserved during surgery, both of which are more important than achieving gross-total removal. Based on these strategies, the authors achieved a favorable outcome rate of 94.7%.

### Postoperative Complications

The two major postoperative complications were hydrocephalus and visual field impairment, both in the authors’ practice and in the literature (**Figure 1** and **Tables 1**, **2**). The rate of preoperative hydrocephalus was considerable, and the condition usually presented with radiographic evidence (3, 5, 6, 12, 14, 15). Some patients had to receive an external ventricular drain or endoscopic third ventriculostomy before the surgery, and in most cases, the condition was relieved after tumor removal. The remaining cases required further V–P shunts to cure the hydrocephalus. In our cases, Ommaya implantation was performed for those pre-operation hydrocephalus conditions. It could be used not only for pre-operative hydrocephalus relief but also for post-operation hydrocephalus temporary therapy.

Regarding visual field impairment, it appeared that fewer cases were reported in our research than in previous studies. Given the advances in anesthesia and related techniques, we can achieve appropriate intracranial pressure control. In addition, we prefer to control intracranial pressure *via* gravity assistance, cerebrospinal fluid release by lumbar puncture, or extraventricular drainage. If necessary, a brain spatula is used to prod the falx to gain more operating space instead of pulling on the occipital lobe.

The hybrid concept, in combination with microsurgery and interventional therapy, is incorporated into the neurosurgical procedure to help lower intraoperative bleeding for complex lesions and reduce iatrogenic damage to the brain parenchyma (case 48). Given the progress in modern anesthesia, the combination of endoscopic and hybrid interventional embolization may be helpful for promoting minimally invasive incision and reducing complications (18).

### Recurrence

Among the nine patients who experienced recurrence in our study, none achieved a Simpson grade I resection. The corresponding pathology showed two cases of angiopericytoma (WHO II) and seven cases of meningioma (WHO I). Six tumor locations were of the superior type, and four of the tumors were calcified. Similar to the literature, recurrences are mainly observed in patients after nonradical resection and/or with atypical or anaplastic meningiomas or hemangiopericytoma (3, 5). Usually, MR contrast images were obtained after operation to make sure the tumor residual, during the follow-up period, and following therapy. Recurrence is the outcome event in this study. For the primary recurrent tumor, craniotomy tumor resection is optimal. The appropriate stopping point comes when it is difficult to remove the tumor totally.

### Limitation

The retrospective nature of this study may have led to a selection bias. A total of 22.4% of patients were lost to follow-up, which may be a confounder that led to the high recurrence rate. Small samples may also induce complication loss, such as seizure attack or sinus injury in our study.

## Conclusion

The OIA is the most commonly used and safest approach for resecting falcotentorial junction tumors with multiple brain pressure control assistance techniques, followed by the SCITA. The selection of surgical approach must be based on imaging features, such as laterality, Asari types, and the presence of a straight sinus occlusion.

## Data Availability Statement

The raw data supporting the conclusions of this article will be made available by the authors, without undue reservation.

## Ethics Statement

The studies involving human participants were reviewed and approved by Huashan Hospital Institutional Review Board. The patients/participants provided their written informed consent to participate in this study.

## Author Contributions

Study conception and design were contributed by WZ and JS. Acquisition of data and follow up was contributed by PL. Surgical participation was rendered by JS, PL, XW, KQ, YL, and WZ. Pathological confirmation was performed by ZY. Drafting of manuscript was done by PL. Critical revision was carried out by all authors. All authors contributed to the article and approved the submitted version.

## Funding

This study was supported by the Shanghai Rising-Star Program (18QA1400900).

## Conflict of Interest

The authors declare that the research was conducted in the absence of any commercial or financial relationships that could be construed as a potential conflict of interest.

## Publisher’s Note

All claims expressed in this article are solely those of the authors and do not necessarily represent those of their affiliated organizations, or those of the publisher, the editors and the reviewers. Any product that may be evaluated in this article, or claim that may be made by its manufacturer, is not guaranteed or endorsed by the publisher.

## References

[1] Bassiouni H Asgari S Konig HJ Stolke D. Meningiomas of the Falcotentorial Junction: Selection of the Surgical Approach According to the Tumor Type. Surg Neurol (2008) 69(1):339–49; discussion 349. doi: 10.1016/j.surneu.2007.02.029 17707469

[2] Bassiouni H Hunold A Asgari S Stolke D. Tentorial Meningiomas: Clinical Results in 81 Patients Treated Microsurgically. Neurosurgery (2004) 55:108–16; discussion 116-8. doi: 10.1227/01.NEU.0000126886.48372.49 15214979

[3] Zhao X Belykh E Przybylowski CJ Borba ML Gandhi S Tayebi MA. Surgical Treatment of Falcotentorial Meningiomas: A Retrospective Review of a Single-Institution Experience. J Neurosurg (2019) 133(3):630–41. doi: 10.3171/2019.4.JNS19208 31374550

[4] Blasco GDAG Delgado-Fernandez J Penanes CJ Gil-Simoes R Frade-Porto N Sanchez MP. Meningiomas Originated at the Falcotentorial Region: Analysis of Topographic and Diagnostic Features Guiding an Optimal Surgical Planning. World Neurosurg (2019) 123:e723–33. doi: 10.1016/j.wneu.2018.12.013 30580064

[5] Talacchi A Biroli A Hasanbelliu A Locatelli F. Surgical Management of Medial Tentorial Meningioma: Falcotentorial and Torcular. World Neurosurg (2018) 115:e437–47. doi: 10.1016/j.wneu.2018.04.066 29678716

[6] Hong CK Hong JB Park H Moon JH Chang JH Lee KS. Surgical Treatment for Falcotentorial Meningiomas. Yonsei Med J (2016) 57:1022–8. doi: 10.3349/ymj.2016.57.4.1022 PMC4951445 27189300

[7] Lensing FD Abele TA Sivakumar W Taussky P Shah LM Salzman KL. Pineal Region Masses–Imaging Findings and Surgical Approaches. Curr Probl Diagn Radiol (2015) 44:76–87. doi: 10.1067/j.cpradiol.2014.05.007 25027864

[8] Rey-Dios R Cohen-Gadol AA. A Surgical Technique to Expand the Operative Corridor for Supracerebellar Infratentorial Approaches: Technical Note. Acta Neurochir (Wien) (2013) 155:1895–900. doi: 10.1007/s00701-013-1844-4 23982230

[9] Odake G. Meningioma of the Falcotentorial Region: Report of Two Cases and Literature Review of Occlusion of the Galenic System. Neurosurgery (1992) 30:788–93; discussion 793-4. doi: 10.1227/00006123-199205000-00027 1584399

[10] Yamazaki T Takahashi S Ishii K Matsumoto K Ishibashi T Sakamoto K. Meningioma in the Pineal Region: Preoperative Diagnosis With CT, MRI, and Angiography. Radiat Med (1991) 9:22–5. 1852901

[11] Behari S Das KK Kumar A Mehrotra A Srivastava AK Sahu RN. Large/Giant Meningiomas of Posterior Third Ventricular Region: Falcotentorial or Velum Interpositum? Neurol India (2014) 62:290–5. doi: 10.4103/0028-3886.136934 25033852

[12] Nowak A Dziedzic T Czernicki T Kunert P Marchel A. Falcotentorial and Velum Interpositum Meningiomas: Two Distinct Entities of the Pineal Region. Neurol i Neurochirurgia Polska (2014) 48:397–402. doi: 10.1016/j.pjnns.2014.09.009 25482250

[13] Asari S Maeshiro T Tomita S Kawauchi M Yabuno N Kinugasa K. Meningiomas Arising From the Falcotentorial Junction. Clin Features Neuroimaging Studies Surg Treat J Neurosurg (1995) 82:726–38. doi: 10.3171/jns.1995.82.5.0726 7714596

[14] Li Y Zhao G Wang H Zhu W Qu L Li Y. Use of 3D-Computed Tomography Angiography for Planning the Surgical Removal of Pineal Region Meningiomas Using Poppen's Approach: A Report of Ten Cases and a Literature Review. World J Surg Oncol (2011) 9:64. doi: 10.1186/1477-7819-9-64 21676231 PMC3125353

[15] Qiu B Wang Y Ou S Guo Z Wang Y. The Unilateral Occipital Transtentorial Approach for Pineal Region Meningiomas: A Report of 15 Cases. Int J Neurosci (2014) 124:741–7. doi: 10.3109/00207454.2013.878341 24397496

[16] Bonney PA Boettcher LB Cheema AA Maurer AJ Sughrue ME. Operative Results of Keyhole Supracerebellar-Infratentorial Approach to the Pineal Region. J Clin Neurosci (2015) 22:1105–10. doi: 10.1016/j.jocn.2014.12.029 25913279

[17] Song J Hua W Pan Z Zhu W. Fully Endoscopic Supracerebellar Infratentorial Approach for Resection of Third Ventricle Germ Cell Tumor: 2-Dimensional Operative Video. Oper Neurosurg (Hagerstown) (2019) 16:389–90. doi: 10.1093/ons/opy150 30010869

[18] Liu JK Cohen MA. Endoscopic-Assisted Posterior Interhemispheric Retrocallosal Transfalcine Approach for Microsurgical Resection of a Pineal Region Falcotentorial Meningioma: Operative Video and Technical Nuances. Neurosurg Focus (2016) 40(videosuppl1):1. doi: 10.3171/2016.1.FocusVid.15453 26722688

