# Supplementary material for: Surgical Management of Falcotentorial Junction Tumors: A Case Series Report
Source: Front Oncol. 2022 May 10;12:866225. doi: 10.3389/fonc.2022.866225 (PMC9128551; doi:10.3389/fonc.2022.866225)
Supplement: Supplementary file 1 [file Table_1.doc]

**Table 1.** **The detailed clinical characteristics of patients with falcotentorial junction tumors**

| **No.** | **Gender** | **Age** | **Clinical Presentation** | **Assari Classification** | **Straight sinus occlusion** | **Laterality** | **Surgical approach** | **Simpson Resection Grading** | **Pathology** | **Major intra-op and post-op complications** | **Follow-up**  **(months)** | **Recurrence** | **GOS** |
| --- | --- | --- | --- | --- | --- | --- | --- | --- | --- | --- | --- | --- | --- |
| 1 | Female | 54 | Headache | Superior | + | Unilateral | Posterior interhemispheric approach | II | Meningioma | None | 10 | - | 5 |
| 2 | Female | 33 | Headache | Posterior | + | Unilateral | Occipital interhemispheric transtentorial approach/ Poppen | II | Hemangiopericytoma | None | 10 | - | 5 |
| 3 | Male | 67 | Dizziness, limb weakness | Inferior | + | Bilateral | Occipital interhemispheric transtentorial approach/ Poppen | II | Hemangiopericytoma | None | 17 | - | 5 |
| 4 | Male | 52 | Blurred vision | Posterior | + | Unilateral | Occipital interhemispheric transtentorial approach/ Poppen | I | Hemangiopericytoma | None | 20 | - | 5 |
| 5 | Female | 34 | Accidentally | Superior | + | Unilateral | Occipital interhemispheric transtentorial approach/ Poppen | II | Hemangiopericytoma | γ-knife | 22 | + | 5 |
| 6 | Male | 56 | Headache | Superior | - | Unilateral | Posterior interhemispheric approach | I | Meningioma | None | 29 | - | 5 |
| 7 | Male | 50 | Blurred vision | Posterior | + | Unilateral | Occipital interhemispheric transtentorial approach/ Poppen | I | Hemangiopericytoma | Contusion | 32 | - | 5 |
| 8 | Female | 51 | Blurred vision | Superior | + | Unilateral | Occipital interhemispheric transtentorial approach/ Poppen | I | Meningioma | None | 40 | - | 5 |
| 9 | Female | 62 | Facial numbness | Superior | + | Bilateral | Occipital interhemispheric transtentorial approach/ Poppen | IV | Meningioma | None | 40 | + | 4 |
| 10 | Female | 47 | Dizziness | Posterior | + | Unilateral | supracerebellar-infratentorial approach | II | Meningioma | None | 41 | - | 5 |
| 11 | Female | 69 | Dizziness | Posterior | + | Bilateral | Occipital interhemispheric transtentorial approach/ Poppen | II | Meningioma | Contusion | 42 | - | 5 |
| 12 | Female | 63 | Headache, Dizziness | Superior | - | Unilateral | Occipital interhemispheric transtentorial approach/ Poppen | II | Meningioma | None | 45 | + | 4 |
| 13 | Female | 60 | Headache, limb weakness | Anterior | + | Bilateral | Posterior interhemispheric approach | I | Meningioma | None | 46 | - | 4 |
| 14 | Female | 69 | Dizziness, unstable gait | Superior | - | Bilateral | Posterior interhemispheric approach | II | Meningioma | None | 49 | - | 5 |
| 15 | Female | 67 | Headache | Anterior | + | Bilateral | Occipital interhemispheric transtentorial approach/ Poppen | II | Meningioma | None | 50 | - | 5 |
| 16 | Female | 58 | Dizziness | Superior | + | Unilateral | Posterior interhemispheric approach | IV | Meningioma | None | 53 | + | 5 |
| 17 | Female | 36 | Headache | Inferior | + | Bilateral | Occipital interhemispheric transtentorial approach/ Poppen | II | Meningioma | None | 53 | - | 5 |
| 18 | Female | 58 | Dizziness | Posterior | ++ | Bilateral | Occipital interhemispheric transtentorial approach/ Poppen | I | Meningioma | None | 54 | - | 5 |
| 19 | Male | 43 | Headache | Posterior |  | Bilateral | Occipital interhemispheric transtentorial approach/ Poppen | II | Meningioma | None | 54 | - | ? |
| 20 | Female | 59 | Headache，dizziness，unstable gait | Anterior | - | Unilateral | Subtemporal | I | Meningioma | None | 57 | - | 5 |
| 21 | Female | 58 | Unstable gait | Inferior | + | Bilateral | supracerebellar-infratentorial approach | II | Meningioma | None | 58 | - | 5 |
| 22 | Female | 40 | Dizziness | Superior | + | Unilateral | Occipital interhemispheric transtentorial approach/ Poppen | I | Meningioma | None | 58 | - | 5 |
| 23 | Female | 62 | Headache, dizziness | Posterior | + | Bilateral | Occipital interhemispheric transtentorial approach/ Poppen | II | Meningioma | None | 58 | - | ? |
| 24 | Female | 42 | Headache | Anterior | + | Bilateral | supracerebellar-infratentorial approach | II | Meningioma | None | 59 | - | 5 |
| 25 | Female | 50 | Headache | Posterior | + | Bilateral | Posterior interhemispheric approach | II | Meningioma | None | 73 | + | 3 |
| 26 | Female | 38 | Dizziness | Posterior | + | Bilateral | Occipital interhemispheric transtentorial approach/ Poppen | I | Meningioma | None | 77 | - | 5 |
| 27 | Female | 60 | Accidentally | Superior | - | Unilateral | Occipital interhemispheric transtentorial approach/ Poppen | II | Meningioma | V-P shunt in 2016 | 78 | + | 4 |
| 28 | Male | 75 | Accidentally | Superior | - | Unilateral | Occipital interhemispheric transtentorial approach/ Poppen | II | Meningioma | V-P shunt in 2019 | 79 | + | 4 |
| 29 | Female | 67 | Dizziness | Superior | + | Unilateral | Posterior interhemispheric approach | II | Meningioma | None | 79 | - | ? |
| 30 | Female | 63 | Facial numbness | Posterior | + | Unilateral | Occipital interhemispheric transtentorial approach/ Poppen | II | Meningioma | None | 80 | - | 5 |
| 31 | Male | 55 | Headache, blurred vision | Anterior | + | Bilateral | Occipital interhemispheric transtentorial approach/ Poppen | II | Meningioma | None | 93 | - | ? |
| 32 | Female | 81 | Headache | Posterior | + | Bilateral | Occipital interhemispheric transtentorial approach/ Poppen | IV | Hemangiopericytoma | Pass away in 2015 | 97 | + | 1 |
| 33 | Male | 60 | Accidentally | Superior | + | Unilateral | Posterior interhemispheric approach | II | Meningioma | None | 102 | - | 5 |
| 34 | Male | 46 | Dizziness | Superior | + | Unilateral | Occipital interhemispheric transtentorial approach/ Poppen | I | Meningioma | None | 103 | - | 5 |
| 35 | Female | 43 | Blurred vision | Anterior | + | Bilateral | Occipital interhemispheric transtentorial approach/ Poppen | I | Meningioma | None | 108 | - | 5 |
| 36 | Male | 49 | Headache, unstable gait | Posterior | + | Bilateral | Occipital interhemispheric transtentorial approach/ Poppen | II | Meningioma | None | 108 | - | 5 |
| 37 | Female | 54 | Unstable gait | Posterior | - | Bilateral | Occipital interhemispheric transtentorial approach/ Poppen | II | Meningioma | None | 112 | - | 5 |
| 38 | Male | 54 | Dizziness, unstable gait | Inferior | - | Bilateral | Occipital interhemispheric transtentorial approach/ Poppen | II | Meningioma | None | 112 | - | ? |
| 39 | Female | 69 | Limb weakness | Anterior | + | Bilateral | Posterior interhemispheric approach | III | Meningioma | None | 115 | + | 5 |
| 40 | Female | 61 | Limb weakness | Posterior | + | Unilateral | Occipital interhemispheric transtentorial approach/ Poppen | II | Meningioma | None | 119 | - | ? |
| 41 | Female | 72 | Accidentally | Inferior | + | Bilateral | Occipital interhemispheric transtentorial approach/ Poppen | I | Meningioma | None | 121 | - | 5 |
| 42 | Female | 72 | Headache, dizziness | Inferior | + | Bilateral | Occipital interhemispheric transtentorial approach/ Poppen | I | Meningioma | None | 123 | - | 5 |
| 43 | Female | 58 | Headache, dizziness | Inferior | + | Bilateral | Occipital interhemispheric transtentorial approach/ Poppen | II | Meningioma | None | 126 | - | 5 |
| 44 | Female | 57 | Headache, dizziness, unstable gait | Inferior | + | Bilateral | Occipital interhemispheric transtentorial approach/ Poppen | II | Meningioma | None | 127 | - | ? |
| 45 | Female | 56 | Epileptic seizure | Anterior | + | Unilateral | Posterior interhemispheric approach | IV | Meningioma | None | 127 | - | 5 |
| 46 | Female | 69 | Headache, dizziness, unstable gait | Anterior | + | Bilateral | Occipital interhemispheric transtentorial approach/ Poppen | II | Meningioma | None | 130 | - | 5 |
| 47 | Female | 65 | Accidentally | Posterior | + | Unilateral | Occipital interhemispheric transtentorial approach/ Poppen | I | Meningioma | None | 144 | - | ? |
| 48 | Male | 55 | Headache | Anterior | - | Bilateral | Occipital interhemispheric transtentorial approach/ Poppen | I | Meningioma | None | 156 | - | ? |
| 49 | Female | 38 | Headache, dizziness | Anterior | - | Bilateral | supracerebellar-infratentorial approach | II | Hemangiopericytoma | None | 164 | - | ? |
